# Association between preconception anti-androgen therapy and pregnancy outcomes of patients with PCOS: A prospective cohort study

**DOI:** 10.3389/fendo.2023.1109861

**Published:** 2023-01-30

**Authors:** Xiaowei Zhang, Huazhang Miao, Jiahe Zhou, Yuan Chen, Yanlan Ou, Yue Song, Xiuhong Peng, Yuancheng Li, Li Li

**Affiliations:** ^1^Department of Obstetrics and Gynecology,Guangdong Women and Children Hospital, Guangzhou, Guangdong, China; ^2^Department of Obstetrics and Gynecology,Dongguan Maternal and Child Health Care Hospital, Dongguan, Dongguan, Guangdong, China; ^3^Guangzhou Medical University, Guangzhou, Guangdong, China

**Keywords:** anti-androgen therapy, polycystic ovary syndrome, adverse pregnancy outcomes, preconception intervention, newborn complications

## Abstract

**Background:**

Polycystic ovary syndrome (PCOS) not only increases fertility challenges for women of reproductive age, but also leads to increased complications during pregnancy and even affects the birth weight of newborns. Also, hyperandrogenemia is associated with lower pregnancy rates and lower live birth rates and may even play a role in preterm delivery and pre-eclampsia in patients with PCOS. However, it is still controversial whether PCOS patients are treated with androgen-lowering therapy before pregnancy.

**Objective:**

To assess the effect of anti-androgen therapy prior to ovulation induction on maternal and infant pregnancy outcomes in patients with PCOS.

**Methods:**

Prospective cohort study.

**Results:**

A total of 296 patients with PCOS were enrolled in the study. The prevalence of adverse pregnancy outcomes, and neonatal complications was lower in DRSP(with drospirenone ethinyl estradiol tablets (II) pretreatment) group than in NO-DRSP(without drospirenone ethinyl estradiol tablets (II) pretreatment) groups (DRSP *vs*. NO-DRSP: adverse pregnancy outcomes, 12.16% *vs*. 27.03%, *P*=0.001; neonatal complications, 17.16% *vs*. 36.67%, *P*<0.001). No significant difference was found in maternal complications. Further subgroup analysis revealed that PCOS with pretreatment decreased the risk of preterm delivery (2.99% *vs*. 10.00%; Adjusted RR, 3.80; 95% CI, 1.19-12.13), pregnancy loss (9.46% *vs*. 18.92%; Adjusted RR, 2.07; 95% CI, 1.08-3.96), low birth weight (0.75% *vs* 7.50%; Adjusted RR, 12.08; 95% CI, 1.50-97.31), fetal malformations(1.49% *vs*. 8.33%; Adjusted RR, 5.63; 95% CI, 1.20-26.33).There were no significant differences in the incidence of DM and PIH as pregnancy complications between the two groups (*P*>0.05).

**Conclusion:**

Our findings suggest that preconception androgen-lowering therapy in patients with PCOS improves pregnancy outcomes and reduces neonatal complications.

## Introduction

Polycystic ovary syndrome (PCOS), the most common complex and heterogeneous endocrine disorder in premenopausal women, has a prevalence of approximately 8%-13% based on foreign studies (1). A survey conducted in 2020, which included 28,739 participants, reported an updated prevalence estimate of 7.8% for PCOS in China (2).

Most women with PCOS experience corresponding problems, such as anovulation, irregular menstruation, and infertility, all of which can lead to a reduced quality of life (3), and an increased incidence of depression and anxiety (4). Therefore, a growing number of studies has focused on PCOS treatment (5) in recent years. Lifestyle changes, including diet, exercise, and behavioral changes, are the first-line treatment recommended for women with PCOS. However, these measures alone have been reported to be ineffective in reducing weight or treating symptoms associated with PCOS (6). Medications are the second-line treatment, and can help to improve pregnancy rates by adjusting hormone levels and improving insulin resistance. In addition to pharmacological interventions, assisted reproductive technologies can also improve pregnancy rates in PCOS patients (7).

PCOS has not only been shown to increase the reproductive burden in women of reproductive age, but it has also been reported to be a risk factor for increased complication rates during pregnancy (8). Complications PCOS patients may experience in early pregnancy include emesis, miscarriage (9). Further risks include gestational diabetes, pre-eclampsia, gestational hypertension, preterm delivery, perinatal fetal death, and increased risk of neonatal intensive care hospitalization (10, 11). With regard to neonatal birth weight, the offspring of PCOS patients have an increased risk of low birthweight babies and oversized babies (11). Despite emerging evidence that PCOS is an unfavorable risk factor for some pregnancies and perinatal outcomes, no guidelines or pharmacological strategies exist for the treatment of PCOS in pregnancy.

Further analysis of some studies has shown that hyperandrogenemia may contribute to ovulatory drug resistance, lower pregnancy rates, and lower live birth rates in patients with PCOS (12), and even play an important role in preterm delivery and preeclampsia (13). The potential impact of hyperandrogenemia on ovulation and pregnancy in PCOS patients suggests that anti-androgen pretreatment may also help to improve fertility in PCOS patients.

Combined Oral Contraceptive (COC) is a class of oral contraceptives that combines estrogen and progestin, and has been used as a first-line treatment for improving hyperandrogenemia and regulating menstrual cycle in PCOS patients of reproductive or adolescent age. Oral COC not only regulates menstrual cycle and reduces androgens in patients with PCOS, but also suppresses hirsutism, treats acne, and prevents endometrial lesions (8). However, controversy persists among researchers as to whether COC should be used before ovulation induction, and whether preconception use of COC improves pregnancy outcomes in patients with PCOS. Palomba et al. reported that COC pretreatment before ovulation induction increased ovulation and pregnancy rates (14), Pan et al. showed that continuous preconception COC interventions not only increased pregnancy rates (14) but also reduced the incidence of small-for-gestational age (15). While a retrospective study by Li et al. found that PCOS patients treated with ethinyl estradiol cyproterone tablets to reduce androgen therapy, followed by ovulation induction, reduced the risk of gestational diabetes mellitus, gestational hypertension-related disorders, and preterm delivery during pregnancy in PCOS patients (16). A randomized controlled trial study by Lergo et al. found that pregnancy rates increased, but live birth rates did not improve in PCOS patients after COC preconception intervention (17). The lack of high-quality evidence on antiandrogen preconception therapy means the effect of COC preconception intervention on pregnancy outcomes in PCOS patients has remained unknown. Consequently, clinicians lack sufficient evidence to convince infertile PCOS patients to spend time and effort to undergo COC preconception treatment before pregnancy, and clinicians continue to disagree on whether to give COC treatment before ovulation promotion and assisted reproductive technology in actual clinical practice.

This study was conducted to settle the aforementioned debate by determining the effect of preconception androgen-lowering treatment intervention on pregnancy outcomes and pregnancy complications in patients with PCOS through a prospective cohort study, and to investigate the need for preconception COC treatment in patients with PCOS. Finally, this study investigates the factors that contribute to adverse pregnancy outcomes in PCOS patients and further help clinicians to recommend possible treatment strategies to prevent adverse pregnancy outcomes in PCOS patients.

## Materials and methods

### Study setting and participants

This is a prospective cohort study with a study population of patients aged 20-35 years with PCOS and fertility needs, who visited the Guangdong Provincial Maternal and Child Health Hospital from September 2019 to April 2022. The study was conducted with the approval of the Ethics Committee of Guangdong Maternal and Child Health Hospital and was successfully registered with the China Clinical Trials Registry (ChiCTR2100052703). All participants gave informed consent prior to enrolling in the study.

Inclusion criteria: (i) meeting the 2003 Rotterdam PCOS diagnostic criteria (2); (ii) PCOS patients aged 20-35 years; (iii) meeting the diagnostic criteria for hyperandrogenemia: elevated total testosterone hormone levels or elevated androstenedione levels (laboratory-defined hyperandrogenemia as total testosterone >1.97 nmol/L and/or androstenedione >10.8 nmol/L).

Exclusion criteria: (1) severe reproductive tract abnormalities; (2) other endocrine disorders, such as diabetes mellitus, thyroid dysfunction and hyperprolactinemia; (3) other systemic diseases, such as cardiovascular, hepatic and renal diseases; (4) malignant tumors; (5) mental challenges that could prevent compliance with treatment or follow-up; (6) the use of any hormones or drugs affecting endocrine and glucolipid metabolism in the 3 months before or during the study period.

### Study cohort

All patients with confirmed PCOS and fertility needs had basic endocrine and glucolipid metabolism examinations on days 3-5 of their menstrual cycle. Anthropometric examinations were performed under the supervision of a professional physician at the time of enrollment. After enrollment, patients in both groups underwent lifestyle modifications under the guidance of professional physicians. Modifications included reducing sugar and fat intake, abstaining from smoking, abstaining from alcohol, and engaging in strengthening exercises. Our exposure factors were 3 consecutive oral cycles of drospirenone and ethinylestradiol tablets (II)(DRSP/EE(II)) before pregnancy in patients with PCOS. Subjects were divided into DRSP group and NO-DRSP group according to whether they voluntarily took DRSP drugs for preconception pretreatment or not. The DRSP group started on the 1st day of the menstrual cycle in the order indicated by the arrows, and 28 consecutive days of oral intake was considered to be 1 complete cycle. Basal endocrine and glucolipid metabolism were rechecked across 3 cycles of treatment. Pregnancy was induced after ovulation immediately after discontinuation of the drug. In the NO-DRSP group, ovulation is induced directly under basic lifestyle guidance. No additional treatment to lower androgen levels was received. Patients were followed up on and monitored for their maternal status, pregnancy complications, delivery and neonatal status.

### Data collection and outcomes

Demographics: age, ethnicity, previous maternal history, height, weight, body mass index (BMI), hip circumference, waist circumference, waist-to-hip ratio (WHR).

Clinical characteristics: All subjects were enrolled with fasting blood sampling for basal endocrine tests, including follicle stimulating hormone (FSH), luteinizing hormone (LH), LH/FSH, estradiol (E2), progesterone (P), serum prolactin (PRL), anti-Müllerian hormone (AMH) on days 3-5 of their menstrual cycles; androgenic parameters: total testosterone (T), androstenedione (AND), dehydroepiandrosterone sulfate (DHEA-S), sex hormone binding globulin (SHBG), free androgen index (FAI); glucose and lipid metabolism indicators: fasting blood glucose (FPG), fasting insulin (FINS), triglycerides (TCH), total cholesterol (TG), low density lipoprotein (LDL), and high density lipoprotein (HDL).

Primary outcome: An adverse pregnancy outcome is a composite indicator that refers to the occurrence of one or more outcomes, such as abortion or preterm labor, during the follow-up window.

Secondary outcomes: Pregnancy complications (gestational diabetes, hypertensive disorders in pregnancy), and neonatal complications (low birthweight babies, oversized babies, neonatal malformations, neonatal referral treatment).

### Statistical analysis

In our study, our main primary outcome was adverse pregnancy outcome, and the DRSP group was designed as a 1:1 cohort study with the NO-DRSP group. The incidence of adverse pregnancy outcomes in the NO-DRSP group was expected to be approximately 39.7%, while the incidence of adverse pregnancy outcomes in the DRSP group was anticipated to be approximately 24.3%. Based on statistical formulae, 143 cases each in the DRSP group and NO-DRSP group were calculated; the follow-up loss rate stayed at about 10%, and the sample size was expanded to 160 cases each in both groups.

Statistical analysis was performed using SPSS 22.0 and GraphPad Prism 8 for graphs. Normality tests were performed on both data sets. Continuous variables that conformed to a normal distribution were expressed as ± SD. Data that did not conform to a normal distribution were expressed as median and quartiles, and categorical variables were expressed as frequencies and percentages. During baseline data collection, missing values were interpolated using single values, where the mean was used for normal continuous variable data, the median for skewed continuous variables, and the plural for categorical variables. Continuous variables were statistically analyzed *via* t-test, while categorical variables were statistically analyzed using Chi-square test or Fisher’s exact probability method. The significance level was determined using a two-sided α = 0.05, when *P* < 0.05 differences were statistically significant.

## Results

### Patient characteristics

**Figure 1:**
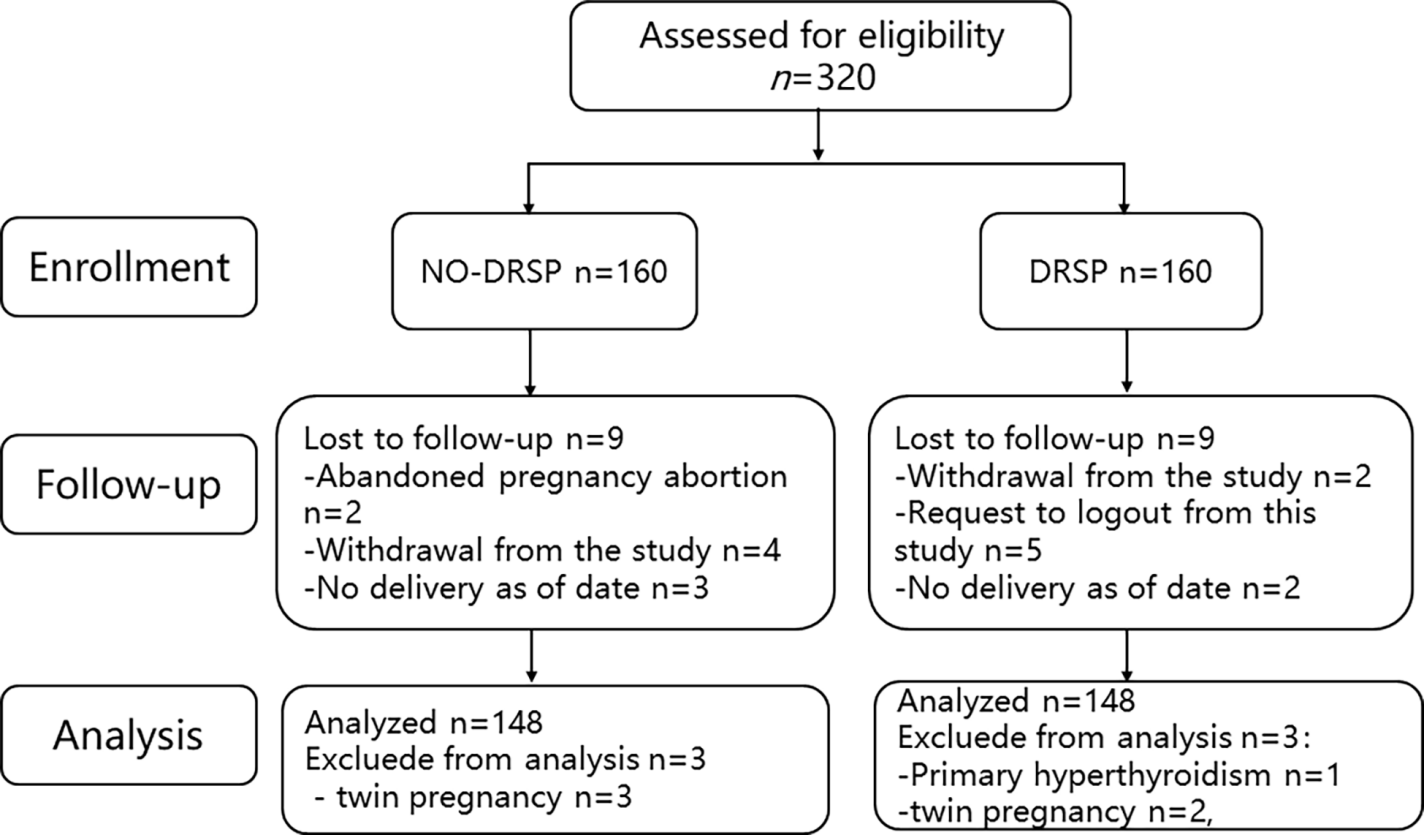
Flow chart of patient registration and follow-up.

As shown in **Figure 1**, a total of 320 pregnant women with PCOS were initially included in our study. During follow-up, three cases were lost in the exposed group, including two cases who requested to withdraw from the study, and one case involving contact error. Four cases were lost in the non-exposed group, including two cases who terminated their pregnancies due to personal factors, and two others who requested to withdraw from the study. A total of 296 patients were included in the final statistical analysis, including 148 patients with PCOS who had undergone pharmacological intervention with drospirenone and ethinyl estradiol tablets (II), and 148 patients with PCOS who had not undergone pharmacological intervention. The clinical and demographic characteristics of the patients are listed in **Table 1**. The results of age, BMI, glucose, lipids, and sex hormone water (*P* > 0.05) at baseline examination were similar in both groups. Overall, the two groups were well matched at the baseline examination.

**Table 1:** Baseline Characteristics of participants.

	NO-DRSP	DRSP	*T*	*P value*
N	148	148		
Age (year)	28.03±2.64	27.53±3.15	3.383	0.067
BMI (kg/m2)	22.24±0.16	22.35±3.22	0.581	0.446
WHR	0.84±0.03	0.85±0.05	33.277	0.000
Pre-BMI (kg/m^2^)	27.93±3.72	26.83±3.19	3.463	0.064
parity				
primipara	139 (91.45%)	119 (79.33%)	8.901	0.003
multiparous	13 (8.55%)	31 (20.67%)		
AMH (ng/ml)	10.54±5.64	11.16±5.57	0.117	0.732
LH (IU/L)	8.25±4.63	9.40±5.08	1.425	0.234
FSH (IU/L)	5.60±1.49	5.38±1.44	0.192	0.661
LH/FSH	1.51±0.91	1.77±0.95	2.707	0.101
T (nmol/L)	1.39±0.53	1.59±0.55	0.160	0.690
AND (mmol/L)	10.53±3.99	12.31±3.99	0.114	0.736
DHEA-S (ug/dl)	272.78±105.81	310.13±111.04	0.016	0.899
FAI	4.57±3.56	4.77±3.39	0.173	0.665
SHBG (nmol/L)	53.34±32.57	46.41±35.36	0.028	0.868
FPG (mmol/L)	4.88±0.34	4.96±0.33	1.057	0.305
FINS (uU/mL)	10.08±1.91	11.37±1.90	11.186	0.003
HOMA-IR (uU/mL)	2.20±0.98	2.42±1.17	6.590	0.011
TC (mmol/L)	4.83±0.87	4.92±0.96	1.222	0.270
TG (mmol/L)	1.49±1.25	1.31±0.76	4.311	0.395
LDL (mmol/L)	4.46±0.87	2.80±0.89	3.194	0.075
HDL (mmol/L)	1.50±1.47	1.39±0.28	2.419	0.121

### Adverse pregnancy outcomes

**Figure 2:**
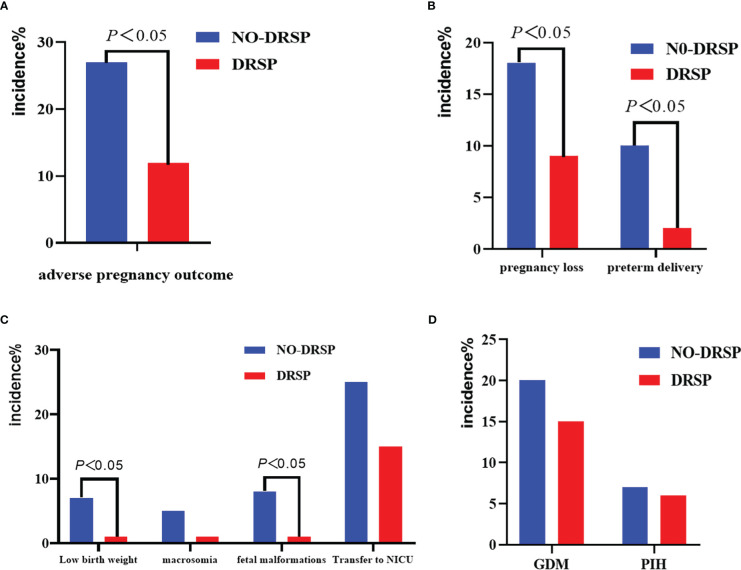
Primary and secondary outcomes in patients with polycystic ovary syndrome (PCOS).

In our study, no postpartum hemorrhage, neonatal asphyxia, or neonatal death events were reported during follow-ups with either group of PCOS patients. As shown in **Table 2** and **Figure 2A**, among 296 subjects, a total of 58 PCOS patients had adverse pregnancy outcome events, including 18 in the DRSP group, and 40 in the NO-DRSP group. The incidence of adverse pregnancy outcomes was significantly lower in PCOS patients treated with DRSP before pregnancy compared to those who were not treated with DRSP, (12.16% *vs*. 27.03%, Adjusted RR, 2.35; 95% CI, 1.34-4.14). In **Table 2** and **Figure 2B**, further subgroup analysis of adverse pregnancy outcomes showed a significant reduction in the incidence of preterm delivery and pregnancy loss in the DRSP group compared with the NO-DRSP group, with a 7.01% reduction in incidence of preterm delivery (2.99% *vs*. 10.00%; Adjusted RR, 3.80; 95% CI, 1.19-12.13) and a 9.46% reduction in incidence of pregnancy loss (9.46% *vs*. 18.92%; Adjusted RR, 2.07; 95% CI, 1.08-3.96).

**Table 2:** Comparison of pregnancy outcomes between two groups. (VS. *DRSP*).

Outcomes	*DRSP n(%)*	NO-DRSP *n(%)*	*Difference (95% CI, %)*	*P value*	*Crude RR (95% CI)*	*P value*	*Adjusted RR (95% CI)*	*P value^a^*
N of sample size	148	148						
Adverse pregnancy outcome								
Adverse pregnancy outcome	18(12.16)	40(27.03)	14.86(5.98~23.75)	0.001	2.22(1.27~3.88)	0.005	2.35(1.34~4.14)	0.003
Pregnancy loss	14(9.46)	28(18.92)	9.46(1.58~17.34)	0.020	2.00(1.05~3.80)	0.034	2.07(1.08~3.96)	0.030
Preterm delivery	4(2.99)	12(10.00)	7.01(0.92~13.11)	0.022	3.35(1.08~10.39)	0.036	3.80(1.19~12.13)	0.024
N of neonates	134	120						
Neonatal complications								
total	23(17.16)	44(36.67)	19.50(8.77~30.23)	<0.001	2.46(1.53~3.94)	<0.001	2.46(1.51~4.01)	<0.001
Low birth weight	1(0.75)	9(7.50)	6.75(1.82~11.69)	0.006	10.05(1.27~79.33)	0.029	12.08(1.50~97.31)	0.019
macrosomia	1(0.75)	6(5.00)	4.25(0.09~8.42)	0.039	6.70(0.81~55.65)	0.078	6.54(0.76~56.52)	0.088
Fetal malformations	2(1.49)	10(8.33)	6.84(1.49~12.20)	0.010	5.58(1.22~25.48)	0.026	5.63(1.20~26.33)	0.028
Transfer to NICU	21(15.67)	30(25.00)	9.33(-0.57~19.22)	0.064	1.59(0.91~2.79)	0.101	1.53(0.86~2.73)	0.150
Maternal complications								
Total	26(19.40)	30(25.00)	5.60(-4.64~15.84)	0.283	1.29(0.76~2.18)	0.344	1.06(0.61~1.85)	0.82
PIH	8(5.97)	9(7.50)	1.53(-4.66~7.72)	0.626	1.26(0.48~3.26)	0.639	1.23(0.45~3.33)	0.686
GDM	21(15.67)	24(20.00)	4.33(-5.11~13.77)	0.367	1.28(0.71~2.29)	0.414	1.12(0.60~2.06)	0.727

a Calibration for age, gestational age, BMI, HOMA-IR index.

### Neonatal complications

A total of 254 newborns were included in our study: 134 in the DRSP group and 120 in the NO-DRSP group. As shown in **Table 2** and **Figure 2C** there was a significant difference in the incidence of neonatal complications in the offspring of PCOS patients in the DRSP group (17.16% *vs*. 36.67%, Adjusted RR, 2.46; 95% CI, 1.51-4.01), where the risk of low birthweight infants was significantly lower in PCOS patients treated with DRSP before pregnancy compared to the control group (0.75% *vs*. 7.50%; Adjusted RR, 12.08. 95% CI, 1.50-97.31). The risk of congenital malformations in the offspring of PCOS patients treated with DRSP before pregnancy was also significantly lower than in the control group (1.49% *vs*. 8.33%; Adjusted RR, 5.63; 95% CI, 1.20-26.33). The incidence of gigantism in the offspring and neonatal treatment referral was similar in both groups of PCOS patients.

### Maternal complications

In **Table 2** and **Figure 2D**, we counted the PCOS patients with severe gestational complications like gestational diabetes mellitus and gestational hypertension-related disorders among the 120 live births in the NO-DRSP group and 134 live births in the DRSP group. Overall, there was no significant difference in the incidence of pregnancy complications in PCOS patients in the DRSP group compared with the NO-DRSP group (19.40% *vs*. 25.00%; Adjusted RR, 1.06; 95% CI, 0.61-1.85), where the incidence of gestational diabetes mellitus and gestational hypertension-related disorders in the DRSP group compared with the NO-DRSP group were not significantly lower (*P >*0.05).

### Subgroup analysis

We performed a further subgroup analysis of factors influencing total adverse pregnancy outcomes and found that the incidence of adverse pregnancy outcomes was significantly reduced by 14.79% in patients with PCOS less than 30 years of age after preconception androgen-lowering therapy. Pre-pregnancy androgen-lowering therapy significantly reduced the incidence of adverse pregnancy outcomes in both primipara and multipara women, but the effect was more pronounced in the multipara women. The findings also showed that PCOS patients with BMI <25 had a significantly lower incidence of adverse pregnancy outcomes after preconception androgen reduction therapy. When the preconception HOMA-IR of the PCOS population was ≥2.69, the risk of adverse pregnancy outcome after preconception androgen-lowering therapy was significantly lower than that of the PCOS population with HOMA-IR <2.69. These results are available in **Table 3**.

**Table 3:** Comparison of total adverse pregnancy outcomes between the two groups in different subgroups (*vs*. DRSP).

	*N*	*DRSP n(%)*	NO-DRSP *n(%)*	*Difference (95% CI, %)*	*P value*	*Crude RR (95% CI)*	*P value*	*Adjusted RR (95% CI)*	*P value*
N	296	148	148						
Age									
<30 years	212	15(13.04)	27(27.84)	14.79(3.95~25.63)	0.007	2.13(1.14~4.01)	0.019	2.22(1.17~4.20)	0.015^a^
≥30 years	84	3(9.09)	13(25.49)	16.40(0.93~31.87)	0.062	2.80(0.80~9.84)	0.108	3.01(0.84~10.84)	0.091^a^
Parity									
primipara	240	14(11.86)	27(22.13)	10.27(0.87~19.66)	0.035	1.87(0.98~3.56)	0.058	1.97(1.02~3.81)	0.044^b^
multipara	56	4(13.33)	13(50.00)	36.67(13.92~59.41)	0.003	3.75(1.22~11.50)	0.021	3.70(1.16~11.78)	0.027^b^
BMI									
<25	240	16(13.68)	34(27.64)	13.97(3.91~24.03)	0.008	2.02(1.12~3.66)	0.020	2.12(1.17~3.87)	0.014^c^
≥25	56	2(6.45)	6(24.00)	17.55(-1.29~36.39)	0.062	3.72(0.75~18.43)	0.108	5.78(1.10~30.43)	0.038^c^
HOMA-IR									
<2.69	205	12(13.04)	29(25.66)	12.62(2.03~23.21)	0.025	1.97(1.00~3.86)	0.049	2.23(1.11~4.48)	0.025^d^
≥2.69	91	6(10.71)	11(31.43)	20.71(3.33~38.10)	0.014	2.93(1.08~7.93)	0.034	2.68(0.97~7.38)	0.057^d^

a Calibration for gestational age, BMI, HOMA-IR index;

b Corrected for age, BMI, HOMA-IR index;

c Corrected age, gestational age, HOMA-IR index;

d Corrected for age, gestational age, BMI.

## Discussion

Our study is a prospective cohort study that included 296 patients with PCOS tracked to pregnancy outcomes. The findings suggest that preconception standardized androgen-lowering therapy with DRSP/EE(II) may not only significantly reduce the incidence of adverse pregnancy outcomes in patients with PCOS, but also benefit the offspring of pregnant women with PCOS by reducing the risk of neonatal complications, although no significant benefit was observed in terms of complications during pregnancy in the study.

Further subgroup analysis of adverse pregnancy outcomes revealed that the incidence of pregnancy loss and preterm delivery were significantly lower in PCOS patients who had used preconceptional androgen-lowering therapy with COC, and that age, BMI, number of births, and preconceptional HOMA-IR were influential factors in the relationship between preconceptional androgen-lowering therapy and adverse pregnancy outcomes in PCOS patients. These findings may be related to the interaction between hyperandrogenemia and insulin resistance as key factors in the pathogenesis of PCOS (18–20), in line with Naver et al.’s cohort analysis, which found that pregnant PCOS patients diagnosed with hyperandrogenemia had a nearly 2-fold increased risk of preterm delivery and preeclampsia (21).

Other literature has also suggested PCOS-related risks in pregnancy. A large cohort study conducted in Sweden by Fornes et al. showed that PCOS patients were at higher risk for miscarriage, preterm birth, and low birthweight babies (22). McDonnell R et al. suggested that polycystic ovary syndrome additionally increases the risk of ectopic pregnancy in women with PCOS (23). Further studies have reported a potential association with embryonic exposure to a hyperandrogenic environment in early pregnancy, which leads to abnormal embryonic development (24), affects placental function, and interferes with normal placental implantation and other causes of early pregnancy loss (25). Our study found that preconception COC treatment significantly reduced the incidence of miscarriage and congenital malformations in the offspring of PCOS patients, probably due to the effective reduction of androgens associated with the regulated use of COC. Early pregnancy loss is associated with endometrial growth in early pregnancy as well, and studies have found high expression of androgen receptors and estrogen receptors in hyperandrogenemic patients with PCOS (25), resulting in an endometrial environment that is not conducive to embryo implantation or placenta formation. Hyperandrogenemia in pregnant women with PCOS can additionally cause a series of changes in the placenta by affecting the function of intravascular trophoblast cells, thereby causing vasculopathy, inflammation, and abnormal chorionic villus development, which can lead to preterm labor and preeclampsia (26). Other relevant studies have shown that the coexistence of hyperandrogenemia with insulin resistance and/or hyperinsulinemia may increase the risk of adverse pregnancy outcomes such as miscarriage, fetal growth abnormalities, GDM, gestational hypertension, preterm delivery and postpartum hemorrhage in women with PCOS (24, 27).

While Li et al. found that the incidence of gestational hypertension-related disorders incidence was significantly reduced after preconception pretreatment with COC in patients with PCOS, GDM (16), our results to-date have not indicated a significant benefit of preconception androgen reduction therapy in pregnant women with PCOS in terms of the occurrence of gestational hypertension or gestational diabetes mellitus during pregnancy. The inconsistency between our findings and those of Li et al. may be related to differences in the inclusion and exclusion criteria of the study population. For example, Li et al. did not exclude multiple pregnancies, and recruited PCOS patients aged 20-40 years, whereas the inclusion criteria for our study specified an age range of 20-35 years and excluded PCOS patients who had experienced multiple pregnancies. Our deeper analysis of factors influencing adverse pregnancy outcomes in both groups of PCOS patients suggested that the occurrence of adverse pregnancy outcomes in PCOS patients was related to age and BMI. Therefore, a randomized controlled study with a large sample is still needed at a later stage to further determine the effect of preconception COC pharmacological intervention on pregnancy complications.

Our findings not only confirm that preconception COC intervention significantly reduces the risk of low birth weight infants in the offspring of PCOS patients, but also demonstrate that preconception intervention does not increase the incidence of congenital malformations in the offspring. The main types of congenital malformations we observed were developmental abnormalities of the nervous system, musculoskeletal system, and urogenital tract system, while other types of developmental abnormalities were not observed in our study. A higher rate of neonatal transfer in PCOS patients compared to the general population has been associated with preterm birth, hypoglycemia, jaundice and respiratory distress syndrome (28). Although our study found that preconception intervention with DRSP/EE(II) though did not significantly reduce the neonatal transfer rate, there may be a diversity of reasons for neonatal referrals. Increased neonatal perinatal mortality has also been reported in pregnant women with polycystic ovary syndrome compared to those without (29, 30). Currently, there have been no reports of neonatal mortality in our study or other studies on preconception interventions for COC.

Based on various reports of the effects of androgens on pregnancy outcomes and our findings suggesting beneficial effects of preconception COC with androgen-lowering therapy, we believe that preconception administration of standardized androgen-lowering therapy should be a standard of care for patients with PCOS.

### Strengths and limitations

A strength of our study is that it is the first prospective cohort study that examines the effect of preconceptional hypoandrogenic therapy on pregnancy outcomes in patients with PCOS. Our study not only followed subjects until the conclusion of their pregnancies and focused on the effect of preconceptional androgen lowering therapy on pregnancy outcome in PCOS patients, but also further stratified analysis and corrected for other influencing factors that may affect adverse pregnancy outcomes. Our study can thus provide evidence-based medical guidance on whether to treat PCOS patients with preconception androgen-lowering therapy with COC, and provide clinicians with possible treatment strategies to reduce adverse pregnancy outcomes in PCOS patients.

However, our study does have some limitations. First, our study was a single-center prospective study. Further multi-center randomized controlled trial validation studies are needed in the future. Furthermore, our study had a short follow-up period for PCOS offspring and only assessed the effect of preconceptional hypoandrogenic treatment with COC drugs on congenital malformations in offspring; it could not clarify the effect of drugs on offspring growth or reproductive health. Some findings were inconsistent with the results after correcting for possible interactions, and the sample size should be expanded for future validation.

## Conclusions

Our study confirms that preconception anti-androgen therapy in patients with polycystic ovary syndrome is effective in reducing both the incidence of adverse pregnancy outcomes as well as the incidence of neonatal complications in the offspring of patients with PCOS. Based on the results of our study, which was a prospective cohort study with strict case-selection criteria, we conclude that preconception androgen-lowering therapy with COC medications in patients with PCOS would be beneficial for pregnancy outcomes and offspring outcomes, thus supporting the standardization of preconception androgen-lowering therapy as a treatment for patients with PCOS.

## Data availability statement

The original contributions presented in the study are included in the article/**Supplementary Material**. Further inquiries can be directed to the corresponding author.

## Ethics statement

The studies involving human participants were reviewed and approved by Medical Ethics Committee of Guangdong Women and Children’s Hospital (NO. 202101273). The patients/participants provided their written informed consent to participate in this study.

## Author contributions

Concept and design: All authors. Acquisition, analysis, and interpretation of data: LL, XZ, HM, JZ, YC, YO, YS, XP, YL. Drafting of the manuscript: XZ, HM. Statistical analysis: XZ, HM. Securement of funding: LL. Administrative, technical, and material support: LL. Supervision: LL. All authors contributed to the article and approved the submitted version.

## Supplementary material

Supplementary Table 1 The occurrence of congenital malformations in the offspring of both groups.

## References

[1] Zeng X Xie YJ Liu YT Long SL Mo ZC. Polycystic ovarian syndrome: Correlation between hyperandrogenism, insulin resistance and obesity. Clin Chim Acta (2020) 502:214–21. doi: 10.1016/j.cca.2019.11.003 31733195

[2] Yang R Li Q Zhou Z Qian W Zhang J Wu Z. Changes in the prevalence of polycystic ovary syndrome in China over the past decade. Lancet Reg Health West Pac (2022) 25:100494. doi: 10.1016/j.lanwpc.2022.100494 35669932 PMC9162959

[3] Teede HJ Misso ML Costello MF Dokras A Laven J Moran L. Recommendations from the international evidence-based guideline for the assessment and management of polycystic ovary syndrome [published correction appears in hum reprod. Hum Reprod (2018) 33(9):1602–18. doi: 10.1093/humrep/dey256 PMC6112576 30052961

[4] De Wilde MA Veltman-Verhulst SM Goverde AJ Lambalk CB Laven JS Franx A. Preconception predictors of gestational diabetes: A multicentre prospective cohort study on the predominant complication of pregnancy in polycystic ovary syndrome. In: Human reproduction, vol. 29. Oxford, England (2014). p. 1327–36. doi: 10.1093/humrep/deu077 24777850

[5] Teede H Deeks A and Moran L. Polycystic ovary syndrome: A complex condition with psychological, reproductive and metabolic manifestations that impacts on health across the lifespan. BMC Med (2010) 8:41. doi: 10.1186/1741-7015-8-41 20591140 PMC2909929

[6] Conway G Dewailly D Diamantikandarakis E. The polycystic ovary syndrome: A position statement from the European society of endocrinology. Eur J Endocrinol (2014) 171:1–29. doi: 10.1530/EJE-14-0253 24849517

[7] Mimouni NEH Paiva I Barbotin AL Timzoura FE Plassard D Le Gras S. Polycystic ovary syndrome is transmitted *via* a transgenerational epigenetic process. Cell Metab (2021) 33:513–30. doi: 10.1016/j.cmet.2021.01.004 PMC7928942 33539777

[8] Jin P Xie Y. Treatment strategies for women with polycystic ovary syndrome. Gynecol Endocrinol (2018) 34(4):272–7. doi: 10.1080/09513590.2017.1395841 29084464

[9] Vrbíková J Cibula D. Combined oral contraceptives in the treatment of polycystic ovary syndrome. Hum Reprod Update (2005) 11(3):277–91. doi: 10.1093/humupd/dmi005 15790599

[10] Haakova L Cibula D Rezabek K Hill M Fanta M Zivny J. Pregnancy outcome in women with PCOS and in controls matched by age and weight. Hum Reprod (2003) 18(7):1438–41. doi: 10.1093/humrep/deg289 12832369

[11] Sugiura-Ogasawara M Sato T Suzumori N Kitaori T Kumagai K Ozaki Y. The polycystic ovary syndrome does not predict further miscarriage in Japanese couples experiencing recurrent miscarriages. Am J Reprod Immunol (2009) 61(1):62–7. doi: 10.1111/j.1600-0897.2008.00662.x 19086993

[12] Qin JZ Pang LH Li MJ Fan XJ Huang RD Chen HY. Obstetric complications in women with polycystic ovary syndrome: A systematic review and meta-analysis. Reprod Biol Endocrinol (2013) 11:56. doi: 10.1186/1477-7827-11-56 23800002 PMC3737012

[13] Xiao Q Cui YY Lu J Zhang GZ Zeng FL. Risk for gestational diabetes mellitus and adverse birth outcomes in Chinese women with polycystic ovary syndrome. Int J Endocrinol (2016) 2016:5787104. doi: 10.1155/2016/5787104 27066074 PMC4808671

[14] Pan JX Liu Y Ke ZH Zhou CL Meng Q Ding GL. Successive and cyclic oral contraceptive pill pretreatment improves IVF/ICSI outcomes of PCOS patients and ameliorates hyperandrogenism and antral follicle excess. Gynecol Endocrinol (2015) 31(4):332–6. doi: 10.3109/09513590.2014.995621 25558892

[15] Rausch ME Legro RS Barnhart HX Schlaff WD Carr BR Diamond MP. Predictors of pregnancy in women with polycystic ovary syndrome. J Clin Endocrinol Metab (2009) 94(9):3458–66. doi: 10.1210/jc.2009-0545 PMC2741722 19509098

[16] Carlsen SM Vanky E. Metformin influence on hormone levels at birth, in PCOS mothers and their newborns. Hum Reprod (2010) 25(3):786–90. doi: 10.1093/humrep/dep444 20023292

[17] Palomba S Falbo A Orio F Jr Russo T Tolino A Zullo F. Pretreatment with oral contraceptives in infertile anovulatory patients with polycystic ovary syndrome who receive gonadotropins for controlled ovarian stimulation. Fertil Steril (2008) 89(6):1838–42. doi: 10.1016/j.fertnstert.2007.05.035 17980363

[18] Negrato CA Jovanovic L Tambascia MA Geloneze B Dias A Calderon Ide M. Association between insulin resistance, glucose intolerance, and hypertension in pregnancy. Metab Syndr Relat Disord (2009) 7(1):53–9. doi: 10.1089/met.2008.0043 18847384

[19] Kriplani A Periyasamy AJ Agarwal N Kulshrestha V Kumar A Ammini AC. Effect of oral contraceptive containing ethinyl estradiol combined with drospirenone vs. desogestrel on clinical and biochemical parameters in patients with polycystic ovary syndrome. Contraception (2010) 82(2):139–46. doi: 10.1016/j.contraception.2010.02.009 20654754

[20] Bates GW Legro RS. Longterm management of polycystic ovarian syndrome (PCOS). Mol Cell Endocrinol (2013) 373(1-2):91–7. doi: 10.1016/j.mce.2012.10.029 PMC4367484 23261983

[21] Naver KV Grinsted J Larsen SO Hedley PL Jørgensen FS Christiansen M. Increased risk of preterm delivery and pre-eclampsia in women with polycystic ovary syndrome and hyperandrogenaemia. BJOG (2014) 121(5):575–81. doi: 10.1111/1471-0528.12558 24418062

[22] Fornes R Simin J Nguyen MH Cruz G Crisosto N van der Schaaf M. Pregnancy, perinatal and childhood outcomes in women with and without polycystic ovary syndrome and metformin during pregnancy: A nationwide population-based study. Reprod Biol Endocrinol (2022) 20(1):30. doi: 10.1186/s12958-022-00905-6 35130922 PMC8819934

[23] McDonnell R Hart RJ. Pregnancy-related outcomes for women with polycystic ovary syndrome. Womens Health (Lond) (2017) 13(3):89–97. doi: 10.1177/1745505717731971 28934902 PMC7789031

[24] Al-Biate MA. Effect of metformin on early pregnancy loss in women with polycystic ovary syndrome. Taiwan J Obstet Gynecol (2015) 54(3):266–9. doi: 10.1016/j.tjog.2013.06.020 26166338

[25] Makieva S Saunders PT Norman JE. Androgens in pregnancy: Roles in parturition. Hum Reprod Update (2014) 20(4):542–59. doi: 10.1093/humupd/dmu008 PMC4063701 24643344

[26] Palomba S Russo T Falbo A Di Cello A Tolino A Tucci L. Macroscopic and microscopic findings of the placenta in women with polycystic ovary syndrome. Hum Reprod (2013) 28(10):2838–47. doi: 10.1093/humrep/det250 23756703

[27] Joham AE Palomba S Hart R. Polycystic ovary syndrome, obesity, and pregnancy. Semin Reprod Med (2016) 34(2):93–101. doi: 10.1055/s-0035-1571195 26854709

[28] Chen Z Tan J Wang H Zheng B Liu J Hao G. A randomized cohort study: Is it worth the time to receive antiandrogenic pretreatment before ovulation induction for women with polycystic ovary syndrome. Front Endocrinol (Lausanne) (2022) 13:813188. doi: 10.3389/fendo.2022.813188 35282449 PMC8907996

[29] Xing C Zhang J Zhao H He B. Effect of sex hormone-binding globulin on polycystic ovary syndrome: Mechanisms, manifestations, genetics, and treatment. Int J Womens Health (2022) 14:91–105. doi: 10.2147/IJWH.S344542 35140526 PMC8818772

[30] Løvvik TS Carlsen SM Salvesen Ø Steffensen B Bixo M Gómez-Real F. Use of metformin to treat pregnant women with polycystic ovary syndrome (PregMet2): A randomised, double-blind, placebo-controlled trial. Lancet Diabetes Endocrinol (2019) 7(4):256–66. doi: 10.1016/S2213-8587(19)30002-6 30792154

