# Supplementary material for: Association between preconception anti-androgen therapy and pregnancy outcomes of patients with PCOS: A prospective cohort study
Source: Front Endocrinol (Lausanne). 2023 Jan 30;14:1109861. doi: 10.3389/fendo.2023.1109861 (PMC9923094; doi:10.3389/fendo.2023.1109861)
Supplement: Supplementary Table 1 — The occurrence of congenital malformations in the offspring of both groups. [file Table_1.docx]

Supplementary materials：

Table 1 The occurrence of congenital malformations in the offspring of both groups.

|  | Cardiovascular | Urogenital | Musculoskeletal | Gastrointestinal | Nervous system | Other |
| --- | --- | --- | --- | --- | --- | --- |
| DRSP | 0 | 0 | 0 | 0 | 2 | 0 |
| NO-DRSP | 1 | 2 | 4 | 0 | 2 | 1 |
| ALL | 1 | 2 | 4 | 0 | 4 | 1 |
